# Does Listening to Mozart benefit Children with Severe Epilepsy?

**DOI:** 10.15844/pedneurbriefs-29-8-2

**Published:** 2015-08

**Authors:** J. Gordon Millichap

**Affiliations:** 1 Division of Neurology, Ann & Robert H. Lurie Children's Hospital of Chicago, Chicago, IL; 2 Departments of Pediatrics and Neurology, Northwestern University Feinberg School of Medicine, Chicago, IL

**Keywords:** Mozart, Music Therapy, Epileptic Encephalopathy

## Abstract

Investigators from the Universities of Salerno and Perugio, Italy, studied the effect of listening to a set of Mozart's compositions on sleep quality, behavior, and seizure recurrence in 11 outpatients (7 males and 4 females), between 1.5 and 21 years of age (mean age, 11.9 years), with drug-resistant epilepsy.

Investigators from the Universities of Salerno and Perugio, Italy, studied the effect of listening to a set of Mozart's compositions on sleep quality, behavior, and seizure recurrence in 11 outpatients (7 males and 4 females), between 1.5 and 21 years of age (mean age, 11.9 years), with drug-resistant epilepsy. All patients had severe intellectual disability and cerebral palsy. They listened to Mozart 2 h per day for 15 days for a total of 30 h. The music was filtered by a device that delivered higher sound frequencies (>3000 Hz). A variety of Mozart musical compositions presented to each patient included symphonies 41 and 46, piano concerto 22 (k482), violin concertos 1 and 4, and flute concerto (k314). During music therapy, 3 patients had a seizure reduction of 75-89%, and 2 out of 11 patients had a reduction of 50-75% in seizure recurrence. The average seizure reduction for all patients was 48.4 +/- 48.7% (p = 0.02). In the two weeks after the end of music therapy, the percentage decrease of total seizure number compared with base-line was 20.7%. The majority (10/11) had multiple EEG foci; occipital/bioccipital discharges were present in 20% of responders and in 66.7% of nonresponders. Responders had an increased frequency of extra occipital EEG discharges and a relative paucity of occipital discharges. Nighttime sleep was improved in 4 (36.4%) patients, all seizure responders. All responders showed improvement in behavior. [1]

COMMENTARY. Previous studies have emphasized the “Mozart effect” of listening to the K448 sonata for two pianos. A wider set of Mozart musical compositions was selected in the present study to improve children's compliance with music listening. The responsiveness of not only seizures but also the EEG interictal discharges is stressed. Generalized and central spike and wave discharges in particular are controlled while listening to Mozart [2]. Very few studies have demonstrated a reduction in clinical seizures in children in response to music, and these are uncontrolled.

Limitations of the present study noted by the authors include the study design (nonrandomized, open-label), and small sample size. Potential strengths include a longer listening period compared to previous studies, and a homogeneous patient sample. Overall, there is limited evidence to recommend Mozart's music as an antiepileptic therapy. The authors find their preliminary findings encouraging and they propose to continue an objective assessment of Mozart as a promising complementary treatment of epilepsy.
